# Rehabilitation After Total Laryngectomy: An Integrated Protocol Remotely Delivered During COVID-19

**DOI:** 10.5195/ijt.2023.6548

**Published:** 2023-05-11

**Authors:** Ylenia Longobardi, Vezio Savoia, Rosa Libero, Maria Elisabetta Marenda, Ilaria Proietti, Pasqualina Maria Picciotti, Giorgia Mari, Claudio Parrilla, Lucia D'Alatri

**Affiliations:** 1 Unità Operativa Complessa di Otorinolaringoiatria, Dipartimento Scienze dell'Invecchiamento, Neurologiche, Ortopediche e della Testa-Collo, Fondazione Policlinico Universitario A. Gemelli, IRCCS, Roma, Italia; 2 Unità Operativa Semplice di Psicologia Clinica-Governo Clinico, Fondazione Policlinico Universitario A. Gemelli, IRCCS, Roma, Italia; 3 Sezione di Otorinolaringoiatria, Dipartimento Testa-Collo e Organi di Senso, Università Cattolica del Sacro Cuore, Rome, Italy

**Keywords:** COVID-19, Psycho-Oncology, Telerehabilitation, Total Laryngectomy, Tracheoesophageal Speech

## Abstract

The aim of this paper was to evaluate the results of an integrated treatment delivered remotely to laryngectomized patients with voice prosthesis. Eighteen laryngectomized patients were treated remotely in groups co-led by a speech therapist and a psychologist (“Online Group”). The results were compared with those of 17 patients (“In-Person Group”) previously studied. The two groups obtained comparable results on all parameters of the INFVo perceptual rating scale, in the DEP, ANX, PHO and HOS areas of the Symptom Check List-90-Revised questionnaire, and in the areas investigated by the WHOQOL-B questionnaire. The “In-Person Group” obtained statistically better results on the Italian Self-Evaluation of Communication Experiences after Laryngeal Cancer questionnaire. Although the in-person treatment favored the acceptance of the new voice and the development of conversational skills, telerehabilitation guaranteed an adequate level of assistance in terms of voice acquisition, prevention of anxiety and depression, and recovery of a good QoL.

Head and neck cancers account for 4% of all tumors. Among these, laryngeal carcinoma is the most frequent. In recent decades the need to improve the patient's Quality of Life (QoL) has meant that the management of laryngeal cancer has been more oriented towards conservative strategies, both surgical (e.g., partial interventions) and non-surgical (e.g., radio-chemotherapy protocols) (Forastiere et al, 2003). Nevertheless, total laryngectomy (TL) remains, for several authors, the gold standard for the control of locally advanced laryngeal cancer (Bussu et al, 2013; Hoffman et al, 2006).

TL is a highly destructive surgery because it interferes with swallowing, breathing, communication, smell, and taste (Longobardi et al., 2020; Noonan & Hegarty, 2010). Impairment of these functions, together with the presence of a permanent tracheostoma, negatively interferes with the QoL and is responsible for the psychological suffering of laryngectomized patients. Several authors have shown that these patients suffer more intense and significant psychological trauma than that experienced by patients with tumours in other areas (Danker et al., 2010; Singer et al., 2011). The most frequent psychological problems that can arise after TL are adaptation and anxiety-depressive disorders, which often cause prolonged rehabilitation times, reduced QoL, and poor adherence to treatments (Perry et al., 2015). The psychological problems experienced by patients can often be such that they may hinder the standard speech therapy rehabilitation protocol.

To face these difficulties, we recently developed a new rehabilitation protocol, which integrates psychological intervention and speech therapy within group sessions (Longobardi et al., 2019). The protocol arises from the need to intervene from the beginning of the rehabilitation path also on the psychological and emotional level. The results of our previous study showed that taking care of the psychological needs of laryngectomized patients during the traditional speech therapy not only facilitates the learning of the alaryngeal speech but also allows an easier adaptation to it, especially in those patients who refuse it and/or delay its acquisition.

In March 2020, the World Health Organization (WHO) announced the COVID-19 pandemic outbreak. COVID-19 disease is a viral respiratory syndrome caused by the SARS-CoV-2 virus. The main symptoms caused by this virus are fever and cough accompanied by breathing difficulties, as well as loss of taste and smell. The virus is transmitted mainly through droplets of saliva and bioaerosols and can have serious consequences especially in the elderly and in the so-called “fragile” subjects (Hennessy et al., 2020) category that also includes patients who have undergone TL. The latter represent a high-risk population due to the post-surgical anatomical changes (e.g., breathing through the tracheostoma) and the frequent presence of comorbidities, among which chronic pulmonary diseases are of particular importance.

In addition to the risks for the patients, there are risks for healthcare professionals. Indeed, the outpatient operative procedures necessary for the follow-up and/or management of laryngectomized patients are classified as high bioaerosols production and therefore at high risk of transmission of SARS-CoV-2 (Longobardi et al., 2021; Parrilla et al., 2020, 2021). To minimize the risk of contagion, a remodelling of outpatient activities provided for the suspension of in-person group therapy for laryngectomized patients. After an initial period, during which we observed a worsening of the psychological conditions of the patients followed (Longobardi et al., 2021), we decided to propose a new way of carrying out group therapy sessions.

Our Institution (third-level hospital) had already recommended that healthcare workers begin to provide as many remote services as possible to help slow the spread of SARS-CoV-2, approved this decision. Therefore, the integrated rehabilitation protocol was remotely delivered using a commercially available videoconferencing platform that had been authorized for use by our Institute.

The present study aimed to verify the effectiveness of the integrated rehabilitation protocol delivered online in terms of acceptance of the alaryngeal speech, psychological well-being, and self-perceived QoL.

## METHODS

### Design

In this case-control study eighteen voice prosthesis laryngectomized patients were enrolled and treated with an integrated approach delivered online. Psychological distress (SCL-90-R), subjective perception of well-being (WHOQOL-B), levels of adaptation to the new speech (I-SECEL) and perceptual quality of the voice (INFVo scale) were evaluated for each patient, before and after treatment. The results were compared with those previously obtained by our group in a trial on an integrated approach used in-person (Longobardi et al., 2019).

### Subjects

In the period between February 2021 and May 2021, 18 patients (14 men and 4 women; mean age 68.87 ± 8.14 years; range 52–80 years), that had undergone TL and placement of a tracheoesophageal (TE) prosthesis at the Fondazione Policlinico A. Gemelli – IRCCS of Rome, were enrolled in the study (“*Online Group*”).

The control group consisted of 17 patients (14 men and 3 women; mean age 65.64 ± 5.83 years; range 54–75 years) enrolled in the original study of the integrated rehabilitation approach after TL (“*In-Person Group*”) (Longobardi et al., 2019).

Therefore, the total sample analysed in this study consisted of 35 subjects (28 men and 7 women; mean age 66.68 ± 6.66 years; range 52–80 years). Eight out of 35 patients underwent salvage TL (in two cases after partial surgery, in six cases after radio-chemotherapy), while 27 patients underwent TL as a first approach.

Of the 27 patients who underwent primary TL, 22 underwent adjuvant therapy (19 radiotherapy and 3 radiochemotherapy).

All patients received an indwelling low-resistance prosthesis (Provox Vega) (Atos Medical AB, Horby, Sweden). In 23 cases, the prosthesis was inserted by a primary puncture and in 12 by a secondary puncture. Data of the two groups of patients are summarized in Table 1.

**Table 1:** Patient Characteristics

	In-Person Group (N = 17)	Online Group (N = 18)
Mean age (years)	65,64 ± 5,83	68.87±8,14
Sex	3 W, 14 M	4 W, 14 M
ISCED 2: Lower secondary education	11/17	10/18
ISCED 3: Upper secondary education	6/17	8/18
Primary TL	12/17	15/18
Salvage TL	5/17	3/18
Neoadjuvant Treatment	4/17	2/18
Adjuvant Treatment	10/17	12/18
Primary TE	10/17	13/18
Secondary TE	7/17	5/18
Average Time from Primary TEP to Group Taking Charge (months)	2,16 ± 1,45	2,32 ±1,24
Average Time from LT and Secondary TEP (months)	4,62 ± 2,83	6,56 ± 1,35
Average Time from Secondary TEP to Group Taking Charge (weeks)	6,21 ± 1,83	1,83 ± 2,56

*Note*. TL : Total Laryngectomy; TE: Tracheo-Esophageal prosthesis; TEP: Tracheo-Esophageal Puncture

### Rehabilitation Protocol

Immediately after hospital discharge, all patients underwent five individual speech therapy sessions on a weekly basis to ensure the first vocal productions. Subsequently, 16 sessions of group therapies were scheduled. The rehabilitation activity was divided into two phases of eight sessions each.

During the first phase, speech therapy was undertaken while the psychologist played a role as a participant observer. TE speakers were instructed about proper cleaning and handling of the prosthesis, and coordination among breathing, digital closing of the tracheostoma, and speech production. At the same time, the psychologist observed the relational dynamics within the group, with the aim of facilitating communication exchanges and the relationships among patients and between the patients and the speech therapist.

In the second phase the psychologist, in the role of mediator and facilitator of communication exchanges, stimulated patients to address issues and experiences related to the illness. In particular, psychological intervention was focused on: management of emotional reactions in response to the trauma of diagnosis and surgery; elaboration of mourning for the loss of the laryngeal voice; acceptance of the alaryngeal voice; processing of feelings like hostility, aggressiveness, and guilt; and work aimed at improving self-esteem. In this phase the speech therapy activities were more focused on communication and interpersonal skills as well as on exercises useful for the improvement of prosody (Longobardi et al., 2019).

The therapeutic setting represented the only difference between the previous study and the present one. Due to the health emergency because of the Sars-CoV-2 virus spread, all the rehabilitation sessions were held using telerehabilitation (TR). TR was administered using a synchronous approach: both therapists and patients sat in front of the PC monitor and had audio/video connections. TR was performed with a 16GB RAM, HD 500GB, i7 computer connected to a microphone and a camera. The sessions were carried out with a bandwidth internet connection of 256 Kbps, resolution of 640 × 480 pixels and 20 fps, through a videoconferencing platform commercially available and authorized by our Institute.

Patients were asked to remain seated in front of the computer, approximately 50 cm from the screen, to reduce sound distortion and maximize visibility. For patients not technically skilled or who found it difficult to access the sessions independently, the presence of a family member was required only at the beginning of therapy.

All patients signed informed consent. The Medical Ethics Committee of the Catholic University of the Sacred Heart approved the study (ID 3181).

### Assessment

A baseline assessment was performed for each patient, after five individual sessions of speech therapy. A post-therapy assessment was performed immediately after the end of 16 sessions of the group therapy. Assessments of both groups were performed in-person and included patient self-ratings and auditory-perceptual evaluations. All participants were given pencil and paper to complete the questionnaires. There was no difference in the administration of the assessment between the “*Online group*” and the “*In-Person group*”. Patients' self-rating was performed by means of the following questionnaires: the Italian Self-Evaluation of Communication Experiences after Laryngeal Cancer (I-SECEL) (Schindler et al., 2013), the Symptom Check List-90-Revised (SCL-90-R) (Sarno et al., 2011) and the World Health Organization Quality of Life Scale-Brief version (WHOQOL-B) (De Girolamo et al., 2020).

### Italian version of the Self-Evaluation of Communication Experiences after Laryngeal Cancer (I-SECEL)

A self-evaluation tool had been translated into Italian and validated from the original questionnaire published by Blood (1993). The questionnaire assesses the level of adaptation to the new speech with the aim of establishing whether the patient needs specific counselling. It consists of 35 items divided into three subscales: General, Environment and Attitude. Patients rate each statement on a 4-point categorical scale (3 = always; 2 = often; 1 = sometimes; 0 = never), referring to the last 30 days. A total score from 0 to 102 and three sub-scores can be obtained. A higher total score indicates greater perceived difficulty with adjustment to the new voice. A mean Total score for well-adjusted patients is 36 ± 12. A score ≥60 suggests the need for specific psychological intervention for acceptance of the new voice.

### Symptom CheckList-90-Revised

The SCL-90-R questionnaire (Derogatis, 1994) is a self-assessment tool, validated and translated into Italian (Sarno et al., 2011), which evaluates a broad spectrum of psychological problems and psychopathological symptoms. Specifically, the questionnaire evaluates 10 different areas: Somatization, Obsessive-Compulsiveness, Interpersonal Sensitivity, Depression, Anxiety, Hostility, Phobic Anxiety, Paranoid Ideation, Psychoticism, and Sleep Disorders. It consists of 90 items; each item can be rated on a 5-point Likert scale, from 1 (“not at all”) to 5 (“very much”). An average score is calculated for each area; a score equal to or greater than one is considered pathological.

### World Health Organization Quality of Life Scale—Brief Version

The WHOQOL-B is a self-assessment questionnaire that explores the subjective perception of health status (The WHOQOL Group, 1994). Validated in different languages, including Italian (De Girolamo et al., 2020), it consists of 26 questions, each of which the patient can answer using a 5-point Likert scale. The areas investigated by the WHOQOL-B questionnaire are Physical Health (PHY), Psychological Health (PSY), Interpersonal Relations (REL), Environment (ENV). The score of each area is obtained by converting the raw scores into composite scores which can range from 0 to 100. The higher the score, the better the self-perceived QoL.

### Perceptual Assessment

The INFVo scale is a tool specifically designed for the perceptual evaluation of substitution voices (Moerman et al., 2006), translated and validated in Italian (Schindler et al., 2013b). The scale examines the following four parameters: overall impression (I), amount of unwanted additive noises (N), fluency (F) and quality of voicing (Vo). For each parameter, the score can range from 0 to 10. Better voice perception is associated with higher scores.

Blind perceptual evaluation, using the INFVo scale was performed on recorded voice samples by two speech therapy students who had no previous experience or exposure to alaryngeal speech. Patients were asked to read aloud the first three sentences (53 words, 107 syllables) of the passage “Il deserto” (Schindler, 1985). Speech samples were recorded with a sampling rate of 22.050 Hz by using a Shure model SM48 microphone (Evanston, IL, USA) positioned at a 45° angle and 20 cm away from the patient's mouth. The recordings were made in a quiet room (ambient noise < 30 dB) and archived in wav format through the Computerized Speech Lab (CSL) Model 4500 (Kay Elemetrics, Lincoln Park, NJ). Each recorded speech sample was made anonymous and distributed to the raters in a random order.

### Statistical Analysis

The MedCalc statistical package, version 12 (Marienkerke, Belgium) was used. The Kolmogorov–Smirnov test was used to evaluate the distribution of the continuous variables examined in the study. The effect of group (In-Person therapy vs. Online therapy) and the time of therapy (pre vs. post therapy) was analysed by bidirectional analysis of variance (ANOVA) for all variables (psychological distress, self-perceived QoL, degree of adaptation to the new speech, and perceptual evaluation of the voice). Bonferroni correction was applied to the entire analysis. The significance level was set at p <0.05.

## RESULTS

### Italian Self-Evaluation of Communication Experiences after Laryngeal Cancer Questionnaire

A significant effect of both time factor (F (1–64) = 34.54, p <0.001) and group factor (F (1–64) = 6.00, p = 0.01) on total scores of I-SECEL was found (Table 2), with a greater improvement in the “In-Person Group”.

Similarly, all the I-SECEL subscales were significantly influenced by both the time and the group factor (p<0.05) (Table 1). In particular, the “In-Person Group” achieved a greater improvement than that obtained by the “Online Group” in each subscale.

**Table 2:** I-SECEL: Degrees of Freedom (dof), F-values and p-values, Pre-and Post-treatment Scores, and Standard Deviations (SD) for Total Score and for the Scores of the Three Subscales (A, G and E)

	dof	Group factor F	Group Factor p	Time Factor F	Time Factor p	Online Group (N=18)		In-Person Group (N=17)	
						Pre-treatment Mean score ± SD	Post treatment Mean score ± SD	Pre-treatment Mean score ± SD	Post-treatmen Mean score ± SD
I-SECEL TOTAL	1–64	34.54	<0.001	6.00	0.01	41.5 ± 22.40	24.37 ± 20.99	49.70 ± 11.01	25.88 ± 9.04
I-SECEL A	1–64	9.94	0.002	9.20	0.003	9.12±8.64	8.25±8.75	17.47±6.72	8.41±3.50
I-SECEL G	1–64	9.98	0.002	59.82	<0.001	17.5±10.54	5.14±3.58	8.70±3.07	6.29±2.86
I-SECEL E	1–64	5.39	0.02	25.16	<0.001	15.75±12.25	11.25±11.63	23.70±6.32	11.17±5.57

### Symptom Checklist-90-Revised Questionnaire

All areas of the SCL-90-R questionnaire were influenced by both the time factor and the group factor. Specifically, the “In-Person Group” showed a significant improvement in all areas of the questionnaire. The “Online Group,” on the other hand, showed an improvement exclusively in the DEP, ANX, HOS and PHO areas. In the remaining six areas, we observed a worsening that, however, did not exceed the clinical cut-off (Table 3).

**Table 3:** SCL-90-R: Degrees of Freedom (dof), F-values and p-values, Pre- and Post-treatment Scores, and Standard Deviations (SD) f All Parameters

	dof	Group factor F	Group Factor p	Time Factor F	Time Factor p	Online Group (N=18)		In-Person Group (N=17)	
						Pre-treatment Mean score ±SD	Post treatment Mean score ±SD	Pre-treatment Mean score ±SD	Post-treatment Mean score ±SD
SOM	1–64	25.65	<0.001	4.49	0.03	0.35±0.31	0.41±0.33	1.07±0.57	0.62±0.40
PARA	1–64	16.78	<0.001	4.49	0.03	0.25±0.39	0.30±0.38	0.84±0.47	0.42±0.39
O-C	1–64	0.18	0.005	5.20	0.02	0.56±0.45	0.61±0.43	0.76±0.43	0.33±0.32
DEP	1–64	0.41	0.03	14.42	<0.001	0.79±0.51	0.62±0.48	1.07±0.64	0.47±0.27
ANX	1–64	3.09	0.001	8.85	0.004	0.47±0.25	0.42±0.31	0.84±0.61	0.36±0.29
SLEEP	1–64	0.09	0.05	5.46	0.02	0.93±0.68	0.96±0.65	1.3±1.10	0.49±0.49
PHO	1–64	7.29	0.008	1.68	0.01	0.10±0.16	0.07±0.15	0.45±0.68	0.24±0.38
PSY	1–64	8.95	0.003	2.87	0.001	0.12±0.21	0.15±0.21	0.38±0.27	0.18±0.23
INT	1–64	2.07	0.03	2.33	0.005	0.35±0.55	0.40±0.48	0.70±0.58	0.34±0.37
HOS	1–65	4.58	0.03	8.18	0.005	0.47±0.04	0.35±0.04	0.78±0.32	0.41±0.10

### World Health Organization Quality of Life Scale—Brief Version Questionnaire

The FIS and PSY areas were influenced by the time factor but not by the group factor (p> 0.05).

As regards to the REL area, no effect was observed of either the time (p> 0.05) or the group factor (p> 0.05). Finally, the AMB area was influenced by both the time and the group factors. Specifically, at the end of treatment the “Online Group” had significantly better scores in this area than the “In-Person Group” (Table 4).

**Table 4:** WHOQOL-BREF: Degrees of Freedom (dof), F values and p values, Mean Scores Before and After Treatment, Standard Deviations (SD) in the AMB Area

	Dof	Group factor F	Group Factor p	Time Factor F	Time Factor p	Online Group (N=18)		In-Person Group (N=17)	
						Pre-treatment Mean score ± SD	Post treatment Mean score ± SD	Pre-treatment Mean score ± SD	Post treatment Mean score ± SD
FIS	1–64	2.70	NS	34.70	0.001	57.87±15.03	69.62±11.41	49.35±15.14	69.29±10.32
PSY	1–64	1.70	NS	7.14	0.009	59.5±16.20	68.12±19.97	52.64±19.57	64.82±18.88
REL	1–64	0.06	NS	3.59	NS	49.25±14.27	51.5±19.81	45.58±20.31	57±14.44
AMB	1–64	6.98	0.01	4.34	0.04	58 ± 12.61	65,75±15.82	54.82± 10.34	56.70±8.78

### INFVo Scale

Results of the INFVo scale showed a statistically significant improvement in all parameters when considering the time factor (p<0.05). Instead, no significant statistical differences were found in relation to the group factor (p<0.05) (Table 5).

**Table 5:** INFVo Scale: Degrees of Freedom (dof), F-values and p-values, Mean scores, and Standard Deviations (SD) of All Parameters

	dof	Group Factor F	Group Factor p	Time Factor F	Time Factor p	Online Group (N=18)		In-Person Group (N=17)	
						Pre-treatment Mean score ± SD	Post treatment Mean score ± SD	Pre-treatment Mean score ± SD	Post-treatment Mean score ± SD
I	1–65	0.06	NS	65.61	<0.001	4.81±1.88	7.81±1.03	4.82±2.17	7.61±1.39
N	1–65	5.43	NS	60.20	<0.001	4.28±3.18	7.54±1.34	3.70±2.27	7.14±1.24
F	1–65	1.81	NS	56.29	<0.001	4.86±2.26	7.74±0.96	4.35±2.11	7.73±1.69
V0	1–65	0.10	NS	27.59	<0.001	6.42±0.90	7.70±0.79	6.4 ±1.47	7.79±1.11

### Discussion

The purpose of this study was to verify the effectiveness of an integrated approach remotely delivered to laryngectomized patients with voice prosthesis. Results obtained by patients in terms of perceptual quality and acceptance of the new voice, psychological well-being, and self-perceived QoL, were compared with those obtained by patients of our previous study in which an in-person treatment was used (Longobardi et al., 2019).

Based on the results of this study, no significant differences emerged between the “In-Person Group” and the “Online Group” on all parameters of the INFVo scale. In fact, the same improvement was found in both groups who achieved comparably good voice quality after treatment.

As regards the acceptance of the new voice (I-SECEL), while all patients in the “Online Group” showed scores below the clinical threshold (Mean 24.37; SD 20.99), the “In-Person Group” had significantly better scores. For the “Online Group” the distance has probably prevented the development of a sense of sharing among the patients. In fact, although both modalities offer patients the opportunity to experience themselves as conversationalists, the difficulties encountered in connection, in favouring turn taking, and in hearing strongly aperiodic voices via audio may have influenced the development of their conversational skills.

It is possible to hypothesize that similar motivations may explain the SCL-90-R results. The second phase of the integrated rehabilitation protocol focused on the reworking of emotional experiences and on the integration of the new voice in the self-image. This requires high conversational skills. In-person therapy may have allowed for a more solid relationship among patients: a sense of mutual help and the ability for each patient to recognize themselves in the other. These latter aspects may have been hampered in online treatment by the presence of a screen that does not favour adequate emotional closeness among patients.

However, it is important to underline that in the areas relating to depression, anxiety, hostility, and phobic disorder, at the end of the therapy, the same improvement was recorded in the “Online Group” as was previously seen in the “In-Person Group.” The improvement in these areas can be explained by the fact that in the context of the integrated rehabilitation protocol, regardless of the way it is carried out, we focus specifically on the experiences related to anxious-depressive symptoms and on favouring the patient's adaptation to the limits and to the difficulties that characterize their new pathological state.

Demonstrating the effectiveness of online treatment, in terms of vocal rehabilitation and prevention of aspects related to worry, despair and fear, the scores obtained in the WHOQOL-BRIEF questionnaire showed a comparable improvement in both groups, both in terms of subjective perception of physical health (FIS) and psychological well-being (PSY).

Finally, results obtained in the AMB area may have been influenced by the COVID-19 pandemic. The significantly better results recorded in the “Online Group” can be explained by the fact that patients, spending most of their time at home, were able to experience the family environment in a more positive way.

Online treatment is appreciated by patients (Dias et al., 2016; Mari et al., 2022) and it is valid from a purely technical point of view, similar to what has been demonstrated by studies carried out in various other areas of speech therapy (Eslami et al., 2020; Mari et al., 2022; Øra et al., 2020). Overall, online treatment can ensure adequate patient care and good functional results, which in our specific case were represented by the acquisition of voice, the prevention of aspects related to anxiety and depression, and the recovery of a good QoL. Rehabilitation delivered by a remote approach has several benefits, as it reduces barriers to access, travel, treatment delays, overall healthcare costs, and allows patients to be treated in their natural environments. However, it is worth considering that audio and voice quality is not always optimal, creating an impediment especially in the case of highly aperiodic voices with the consequence that neither the therapist nor the patient can have adequate vocal feedback. In the light of what emerged from our study, it seems desirable to carry out further investigations, using the Internet as an operational tool, to deepen the question in the future and promote the improvement of technological resources at the service of clinicians.

## Data Availability

The data that support the findings of this study are available from the corresponding author, C.P. upon reasonable request.
